# Targeting HER proteins in cancer therapy and the role of the non-target HER3

**DOI:** 10.1038/sj.bjc.6603910

**Published:** 2007-07-31

**Authors:** A C Hsieh, M M Moasser

**Affiliations:** 1 Department of Medicine, University of California, San Francisco, San Francisco, CA 94143, USA; 2 UCSF Comprehensive Cancer Center, University of California, San Francisco, San Francisco, CA 94143, USA

**Keywords:** EGFR, HER2, HER3, ErbB3, PI3K/Akt, tyrosine kinase inhibitor

## Abstract

Members of the human epidermal growth factor receptor (HER) family have been of considerable interest in the cancer arena due to their potential to induce tumorigenesis when their signalling functions are deregulated. The constitutive activation of these proteins is seen in a number of different common cancer subtypes, and in particular EGFR and HER2 have become highly pursued targets for anti-cancer drug development. Clinical studies in a number of different cancers known to be driven by EGFR or HER2 show mixed results, and further mechanistic understanding of drug sensitivity and resistance is needed to realise the full potential of this treatment modality. Signalling in trans is a key feature of HER family signalling, and the activation of the PI3K/Akt pathway, so critically important in tumorigenesis, is driven predominantly through phosphorylation in trans of the kinase inactive member HER3. An increasing body of evidence shows that HER3 plays a critical role in EGFR- and HER2-driven tumours. In particular, HER3 lies upstream of a critically important tumorigenic signalling pathway with extensive ability for feedback and cross-talk signalling, and targeting approaches that fail to account for this important trans-target of EGFR and HER2 can be undermined by its resiliency and resourcefulness. Since HER3 is kinase inactive, it is not a direct target of kinase inhibitors and not presently an easily drugable target. This review presents the current evidence highlighting the role of HER3 in tumorigenesis and its role in mediating resistance to inhibitors of EGFR and HER2.

## HER FAMILY

The human epidermal growth factor receptor (HER) family of proteins consisting of EGFR, HER2, HER3, and HER4 are type I transmembrane growth factor receptors that activate intracellular signalling pathways in response to extracellular signals. They consist of an intracellular tyrosine kinase domain, a transmembrane domain, and a glycosylated extracellular Ligand-binding domain. These receptors activate numerous downstream pathways in response to extracellular ligands, regulating diverse processes including differentiation, migration, proliferation, and survival. A distinguishing characteristic of this family is an interdependence on each other and complementarity in their functions. Indeed lateral signalling among the HER proteins constitutes a significant aspect of their signalling functions. Of the four members, HER2 and HER3 are particularly dependent proteins, as they are functionally incomplete receptors by themselves. HER2 has an extracellular domain, but appears to lack ligand-binding activity, while HER3 has a non-functional kinase domain and has no catalytic kinase activity. However, in complementation, the HER2-HER3 heterodimer is a highly functional signalling unit and constitutes the most active signalling dimer in this family, exemplifying the role of complimentary functions in this complex receptor family (Tzahar *et al*, 1996).

The mutational activation of HER family members is seen in many human malignancies. EGFR is found to be overexpressed in more than 80% of head and neck cancers, activated by amplification and/or mutation in approximately 50% of gliomas, activated by mutation in about 10–15% of non-small cell lung cancers (NSCLC) in the west, and 30–50% of NSCLCs in Asia (Frederick *et al*, 2000; Riely *et al*, 2006). Amplification and overexpression of HER2 is seen in about 25–30% of breast cancers and occasionally in other cancers (Slamon *et al*, 1989). The mutational activation of HER2 is also seen in some cancers of the lung (Stephens *et al*, 2004). In contrast to the other family members, the current evidence suggests that HER4 mediates antiproliferative effects; and consistent with this, HER4 overactivity does not appear to play a major role in cancer pathogenesis (Sartor *et al*, 2001; Naresh *et al*, 2006).

## HER2-DRIVEN BREAST CANCER

The best-studied oncogenic model in the HER family has been HER2-induced breast cancer. The HER2 gene is amplified and overexpressed in about 25% of breast cancers, conferring a more aggressive biology (Slamon *et al*, 1987). The aetiologic role of HER2 in mammary tumorigenesis has been confirmed by numerous mouse models of constitutively activated HER2 (or its rodent counterpart Neu) (Ursini-Siegel *et al*, 2007; Moasser, 2007a). HER2 overexpression is potently transforming in numerous cell culture models and in numerous transgenic mouse models (Moasser, 2007a). The experimental evidence also suggests that the overexpression of HER2 is not only aetiologically important in the development of mammary carcinoma, but it continues to drive cancer progression and metastasis in the advanced stages of cancer. Knockdown of HER2 in HER2-overexpressing cancer cells induces apoptotic cell death (Roh *et al*, 2000). In mouse models, wherein metastatic mammary carcinomas are induced by the doxycycline induction of Neu overexpression, withdrawal of the Neu oncogene by cessation of doxycycline therapy results in complete regression of the mammary tumours and its associated tumour metastases (Moody *et al*, 2002). While the simplicity of each of these model systems understates the complexity of HER2-overexpressing human breast cancers, it makes a compelling case that HER2 plays a dominant role in causing and maintaining the transformed phenotype, thus obligating these model systems to oncogenic stimulation. The apparent dependency of HER2-overexpressing tumours on continued HER2 function throughout the natural course of this cancer has made HER2 an attractive target for anti-cancer drug development.

## MECHANISM OF HER2 TUMORIGENESIS: THE ROLE OF HER3

The downstream signalling pathways through which HER2 mediates its tumorigenic functions are complex and have been reviewed recently (Moasser, 2007a). But importantly, in its overexpressed and oncogenic state, HER2 does not appear to escape its dependency on its HER family partners. In particular, an abundance of evidence reveals that its intimate signalling partner HER3 plays an important and necessary function in HER2-mediated tumorigenesis. HER3 is frequently overexpressed in breast cancers with EGFR or HER2 overexpression (Naidu *et al*, 1998). Tumours arising in mice due to the transgenic overexpression of Neu are also characterised by increased expression and phosphorylation of HER3 (Siegel *et al*, 1999). Increased expression of HER3 synergistically increases the transforming potency of HER2 (Alimandi *et al*, 1995). Conversely, loss of HER3 abolishes the transforming ability of HER2, and indeed HER3 is in fact an obligate partner in HER2-mediated transformation (Holbro *et al*, 2003).

The primary oncogenic signalling apparatus afforded to HER2 by its transphosphorylation of HER3 is the PI3K/Akt pathway. While HER2 is unable to directly bind PI3K and activate the PI3K/Akt pathway, the HER2–HER3 signalling complex is highly effective in doing so. This function is directly mediated through HER3, which unlike EGFR and HER2, has six tyrosine containing binding sites for p85, the regulatory subunit of PI3K (Prigent and Gullick, 1994; Soltoff *et al*, 1994). The growth factor activation of PI3K and Akt by HER2 is mediated through the tyrosine phosphorylation of HER3 (Soltoff *et al*, 1994). Although immunohistochemical reagents are currently unable to directly assay HER3 activity in fixed tissues from clinical studies, the activation of HER3 in the mouse model of HER2-induced tumours is established (Siegel *et al*, 1999). Akt activity, however, can be assayed in clinical samples, and the clinical data indeed shows frequent activation of Akt in HER2-expressing tumours (Tokunaga *et al*, 2006a). The role of Akt as a critical proliferative and antiapoptotic signal as well as its contributions to cell invasiveness and metastasis are beyond the scope of this review. However, a plethora of evidence confirm that Akt regulates vital cell functions including proliferation, survival, glucose metabolism, epithelial–mesenchymal transition, genome stability, and angiogenesis. As such, the HER2-HER3 heterodimer activates a pathway that lies in the crossroads of oncogenic signalling and is widely found to be activated in many types of human cancers.

The role of EGFR in HER2-mediated transformation is less apparent. EGFR is not always expressed in HER2-amplified breast cancers and HER2 is fully capable of transforming EGFR-negative cell models (Chazin *et al*, 1992).

## TARGETING HER PROTEINS

The evidence implicating EGFR and HER2 in cancer pathogenesis has driven the development of numerous pharmaceutical approaches to treat cancers using drugs that target EGFR or HER2. These agents have been widely tested in numerous cancers with mixed and sometimes unexpected results. While they have clinical efficacy that is consistent with the scientific evidence showing the critical role of their targets in some cancer subtypes, such as certain lung cancers, they also appear to show very limited efficacy despite compelling scientific evidence predicting otherwise, as in the case of breast cancer. In other cancer subtypes such as colon cancers EGFR-targeting agents also show clinical efficacy. However, a mechanistic basis for this activity is difficult to explain, since EGFR activation is not commonly seen in colon cancers, and the clinical activities of EGFR-targeted therapies shows no correlation with EGFR expression (Chung *et al*, 2005).

## TARGETING HER PROTEINS IN BREAST CANCER

Two modes of targeting HER2 have been extensively studied in the treatment of HER2-overexpressing breast cancer, both *in vitro* and clinically. These include the monoclonal anti-HER2 antibody trastuzumab and several small molecule tyrosine kinase inhibitors (TKIs). Trastuzumab induces tumour regression in approximately 30–35% of patients with HER2-amplified metastatic breast cancer if used as up-front therapy and much less activity if used after other chemotherapies (Tokunaga *et al*, 2006b). Understanding resistance to trastuzumab has been a challenging problem, and this is largely due to the fact that the mechanisms of response to trastuzumab are not well understood. Trastuzumab binds to the extracellular domain of HER2, but extensive efforts have not been able to determine how and whether this suppresses oncogenic HER2 function (reviewed in Moasser, 2007b). Immunologic targeting may play a significant part in the mechanism of action of trastuzumab.

The small molecule TKIs have provided a more mechanistically reliable approach to suppress oncogenic HER2 function. Clinical efficacy studies have been reported with the EGFR-selective erlotinib and gefitinib, the EGFR/HER2 selective lapatinib, and the pan-HER selective canertinib. These agents show only limited activity as single agents in the treatment of HER2-overexpressing breast cancer, despite a preponderance of experimental evidence suggesting that these cancers are highly dependent on HER2 function (Burstein *et al*, 2004; and reviewed in Moasser, 2007b). Correlative scientific data from tumour biopsies in some of these studies confirm that TKIs reach their molecular targets and suppress the activity of EGFR and HER2 and downstream MAPK signalling (Spector *et al*, 2005). Importantly, the inactivation of Akt signalling is not clearly apparent in these studies, suggesting that oncogenic HER2 signalling is not completely suppressed by these therapies. The fact that most HER2-overexpressing breast cancers fail to respond to TKI therapy is somewhat unexpected, given the highly abundant and compelling data that shows that these tumours are driven by and dependent on HER2 function. This has driven new lines of investigation to determine mechanisms by which HER2-overexpressing tumours evade drug therapy.

## ROLE OF HER3 IN BREAST CANCER DRUG RESISTANCE

**Figure 1:**
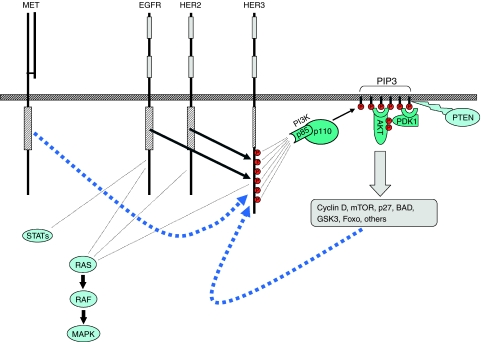
Schematic depicting the central role of HER3 in mediating PI3K/Akt signalling downstream of the HER family. Although all three receptors can bind Grb2 and activate the Ras-Raf-MAPK pathway, only HER3 can activate the PI3K/Akt pathway directly. EGFR and HER2 do not directly bind PI3K, HER3 has six binding sites for PI3K. When phosphorylated by EGFR or HER2, these HER3 phosphotyrosines bind the p85 subunit of PI3K leading to activation of membrane phosphoinositides to phosphoinositol triphosphate (PIP3). PIP3 recruits PH domain containing proteins Akt and PDK1 leading to activation of Akt. PIP3 phosphorylation is negatively regulated by the phosphatase PTEN. Activated Akt effects a diverse range of cellular programs involved in the cell cycle, protein translation, survival, nutrient sensing and metabolism, and gene transcription. Although HER TKIs can suppress the EGFR- or HER2-mediated phosphorylation of HER3 and inactivate downstream Akt signalling, HER3 appears to be a resilient node in this circuitry. Two mechanisms that have been shown to allow HER3 phosphorylation to escape HER TKI therapy are Akt-driven negative feedback signalling that can amplify HER3 signalling, and amplification and overactivity of the tyrosine kinase receptor c-MET that can restore HER3 signalling through crosstalk. These two pathways that are known to mediate HER3 escape from TKI therapy are shown in blue dashed lines.

Recent findings in cell-based studies give new insight to the mechanisms underlying TKI resistance in HER2-driven breast cancers. Tyrosine kinase inhibitors effectively prevent autophosphorylation of EGFR and HER2 in these tumour cells; however, the transphosphorylation of HER3 is only transiently suppressed and HER3 ultimately escapes inhibition by TKIs in HER2-overexpressing tumour cells (Sergina *et al*, 2007). The consequence of HER3 resistance is PI3K/Akt pathway resistance, tumour survival, and escape from the proapoptotic consequences of the loss of oncogenic HER2 signalling. The mechanism that underlies HER3 resistance in these tumours appears to be a forward shift in the phosphorylation-dephosphorylation equilibrium steady state of HER3 signalling, in effect buffering HER3 against an incomplete inhibition of HER2 kinase. The resiliency of HER3 is driven by Akt-mediated negative feedback signaling (Figure 1). The failure to durably suppress HER3 significantly attenuates the anti-tumour activities of TKIs and is entirely consistent with the limited clinical activities of these agents in the treatment of HER2-amplified breast cancers. This study also showed that HER2-driven tumours ultimately cannot escape the total inactivation of HER2 (Sergina *et al*, 2007), leaving hope that more potent TKIs or higher doses of TKIs may yet show significant activity in this disease. This work also reveals an important and unique attribute of the HER2–HER3 signalling complex. The fact that catalytic kinase activity and signalling activity are embodied within different members in this signalling complex confers upon it an extra level of regulation. The additional level of regulation makes it considerably more difficult to suppress HER2 oncogenic signalling with TKIs. The HER2–HER3 complex presents a considerable challenge to drug therapy, since in this complex, HER3 is the principle mediator of resistance, yet HER3 is kinase inactive and is not a direct target of TKIs. In fact, since the signalling function of HER3 is mediated through its role as a substrate of HER2 and a scaffold for recruitment of signalling proteins, it presents a difficult target to inactivate by current pharmaceutical approaches.

## TARGETING HER PROTEINS IN NSCLC

The broad testing of EGFR-selective TKIs in cancers revealed a small subset of highly responsive lung cancer patients (Fukuoka *et al*, 2003). Subsequent studies have identified this subset as patients with cancers driven by mutationally activated EGFR (Lynch *et al*, 2004). Lung cancers driven by mutated EGFR are highly responsive to TKI therapy (Jackman *et al*, 2006). The acquisition of TKI resistance in these patients appears to correlate, in part, with the development of kinase domain mutations that confer resistance to TKI therapy (Kobayashi *et al*, 2005). The identification of EGFR mutations in lung cancer has led to an explosion of interest in studying the mechanisms underlying lung cancer pathogenesis, focusing on the role of HER family proteins. An important role for the kinase-inactive HER3 has also emerged in this disease. Characterisation of lung cancers with TKI-sensitive EGFR mutations reveals that PI3K/Akt pathway in these tumours is dependent on HER3 signalling (Engelman *et al*, 2005). This is analogous to breast cancers with HER2 overexpression, which also depend on HER3 signalling for the activation of PI3K/Akt signalling, discussed above. Tyrosine kinase inhibitor therapy of TKI-sensitive lung cancers inactivates HER3 signalling and downstream PI3K/Akt signalling, but HER3 signalling in TKI-resistant lung cancers appears to be uncoupled from EGFR and resistant to inactivation by TKIs (Engelman *et al*, 2005). The development of the EGFR T790M mutation in lung cancers confers drug resistance and is associated with persistent activation of HER3/PI3K/Akt signalling (Engelman *et al*, 2006). The constitutive activation of HER3 signalling in TKI-resistant lung cancers can also be mediated through the amplification of MET, recently identified in certain TKI-resistant subclones of lung cancer cells (Engelman *et al*, 2007) (Figure 1). Thus the critical role of HER3 as a mediator of the oncogenic functions of the HER family is gradually but surely coming to light in the case of lung cancers.

## TARGETING HER PROTEINS IN MALIGNANT GLIOMAS

EGFR is overexpressed in 40–50% of glioblastoma multiforme (GBM). Of these tumours, nearly half express the EGFRvIII mutation, which is an in-frame deletion of exons 2–7 from the extracellular domain that leads to a constitutively activated receptor (Shinojima *et al*, 2003). Barriers to effective drug biodistribution in the CNS compartment make this disease more challenging for systemic therapy modalities. But there is evidence from phase I studies that HER family TKIs are active in the treatment of this disease (Prados *et al*, 2006). Molecular determinants of treatment response have been studied in patients with GBM. The deletion of PTEN is common in GBMs and correlates with resistance to TKI therapy, and conversely the co-expression of PTEN with EGFRvIII identifies erlotinib-responsive patients (Mellinghoff *et al*, 2005). The effect of PTEN deletion in this pathway is the constitutive activation of downstream Akt signalling. By removing the negative regulation of phosphoinositide signalling, PTEN deletion uncouples HER3 from the Akt pathway, rendering HER family-driven tumours resistant to HER TKIs. This has been demonstrated through the restoration of TKI sensitivity by inducible re-expression of PTEN in PTEN-null tumour cells (She *et al*, 2003). Since the inactivation of PTEN activates Akt signalling at a point downstream of HER3, a role for HER3 in mediating drug resistance in PTEN-deleted tumours may be redundant. The role of HER3 in gliomagenesis and in response and resistance of GBMs to TKI therapy awaits more studies.

## FUTURE DIRECTIONS IN TARGETING HER PROTEINS

Much of the studies of the past decade have focused on the oncogenic functions inherent in the deregulated catalytic kinase activities of EGFR and HER2. Signalling in trans is a key feature of HER family signalling, and although this attribute of HER family signalling has been appreciated for a number of years, the continued dependency of tumours on HER family trans-signalling is just now becoming more apparent. In particular, the emerging role of HER3 in mediating *de novo* or acquired drug resistance has shifted the focus on this critically important lateral signalling partner of EGFR and HER2 and its pivotal role in driving PI3K/Akt signalling. The increasing awareness of the role of HER3 in cancer progression and drug resistance has two implications for future directions. First of all, the correlation of HER3 signalling with response and resistance suggests that HER3 is a much more suitable biomarker to guide the efficacy of treatments compared with more current studies that rely mostly on the autophosphorylation activities of EGFR or HER2. Second, the identification of HER3 as a focal point in response and resistance to TKI therapy identifies it as a novel target for newer anti-cancer agents that can potentially overcome TKI resistance. However, HER3 is a considerably challenging target for pharmaceuticals. Unlike EGFR or HER2, it lacks catalytic kinase activity and its signalling functions cannot be inhibited by TKIs and it is not a direct target of this class of agents. Its signalling functions do include ligand binding, and although antibody therapeutics can be developed to bind its extracellular domain and interfere with ligand binding, it is not clear whether the oncogenic functions of activated EGFR or HER2 are driven by extracellular ligands. The principle signalling function of HER3 in cancers appears to be its role as a substrate of EGFR or HER2 and a scaffold for the recruitment of cytosolic signalling proteins. Targeting scaffold functions remain a challenging goal for current pharmaceutical technologies. The more realistically drugable targets are the ones immediately downstream of HER3. Since the weight of current evidence suggests that the predominant oncogenic function of HER3 is its ability to activate PI3K and Akt signalling, then it is a reasonable hypothesis that inhibitors of PI3K or Akt can also abrogate HER3 signalling and can potentially be used in combination with HER TKIs to develop much more effective therapies or to overcome HER TKI resistance. However, since PI3K and Akt mediate many signalling pathways important for numerous cellular functions in normal tissues, it remains to be determined whether these agents have sufficiently wide therapeutic index to be used in the treatment of cancer. The development and testing of such agents are actively being pursued in the pharmaceutical sector.

Although EGFR and HER2 have been viewed as prototype oncogenes that can drive tumorigenesis through their constitutive activation in cancers, evidence is mounting that their trans-signalling functions are essential aspects of their oncogenic functions. This has highlighted the role of their most active trans-signalling partner HER3. The ability of HER3 to escape drug therapy, its resiliency and resourcefulness, and the multitude of feedback and crosstalk mechanisms that appear to come into play to ensure persistent tumour HER3 signalling activity despite the suppression of EGFR or HER2 by targeted therapies identifies HER3 as a focal point in HER family-induced transformation and a new biomarker and target for future cancer therapies.
